# The potential for linking cohort participants to official criminal records: a pilot study using the Avon Longitudinal Study of Parents and Children (ALSPAC)

**DOI:** 10.12688/wellcomeopenres.16328.1

**Published:** 2020-11-17

**Authors:** Andy Boyd, Alison Teyhan, Rosie P. Cornish, Jazz Croft, Richard Thomas, Iain Brennan, John Macleod

**Affiliations:** 1 School of Population Health Sciences, University of Bristol, Bristol, BS8 2BN, UK; 2 MRC Integrative Epidemiology Unit, University of Bristol, Bristol, BS8 2BN, UK; 3 Department of Criminology and Sociology, University of Hull, Hull, HU6 7RX, UK

**Keywords:** Criminal conviction, official caution, Ministry of Justice, Police National Computer database, record linkage, birth cohort, ALSPAC

## Abstract

**Introduction**: Linking longitudinal cohort resources with police-recorded records of criminal activity has the potential to inform public health style approaches and may reduce potential sources of bias from self-reported criminal data collected by cohort studies. A pilot linkage to police records in the Avon Longitudinal Study of Parents and Children (ALSPAC) allows us to consider the acceptability of this linkage, its utility as a data resource, differences in self-reported crime according to consent status for data linkage, and the appropriate governance mechanism to support such a linkage.

**Methods**: We carried out a pilot study that linked data from the ALSPAC birth cohort to Ministry of Justice (MoJ) records on criminal cautions and convictions. This pilot was conducted on a fully anonymous basis, meaning we cannot link the identified records to any participant or the wider information within the dataset. Using ALSPAC data, we used summary statistics to investigate differences in self-reported criminal activity according to socio-economic background and consent status. We used MoJ records to identify the geographic and temporal concentration of criminality in the ALSPAC cohort.

**Results**: We found that the linkage appears acceptable to participants (4% of the sample opted out), levels of criminality are high enough to support research and that the majority of crimes occurred in Avon & Somerset (the policing area local to ALSPAC). Both those who opted out of linkage or did not respond to consent requests had higher levels of self-reported criminal behaviour compared to participants who provided explicit consent.

**Conclusions**: These findings suggest that data linkage in ALSPAC provides opportunities to study criminal behaviour and that linked individual-level records can provide robust research in the area. Our findings also suggest the potential for bias when only using samples that have explicitly consented to data linkage, highlighting the limitations of opt-in consent strategies.

## Introduction

Policing in the UK increasingly seeks to take a public health approach to tackling crime, where the focus is on proactive prevention, the tackling of upstream risk factors, and on populations rather than individuals ^1^ and emphasising that joint agency approaches are needed ^2^. This approach is multi-disciplinary, and relies on ‘the skilled use and interpretation of data and the evidence base to ensure that interventions are designed, delivered and tailored to be as effective as possible’ ^1^. This can now be seen in operation within some UK police forces – for example, within Thames Valley Police ^1^ – and epidemiological analysis is an important approach to identifying risk and protective factors for criminal and antisocial behaviours. Police records of criminality (e.g. convictions and cautions) do not contain data relating to an individual’s exposure to potential risk factors for perpetrating crime, whereas longitudinal birth cohort studies have a wealth of data on the lives of their participants, and often their families, peers, and wider contexts across the life course. Therefore, linking police data with cohort studies has the potential to add considerable value to research on criminal behaviour (e.g., McAra and McVie 2016 ^3^), providing that measures of the participants’ criminal behaviours are accurate.

One way of obtaining such measures is to use self-report measures of criminal data from participants or related individuals (e.g. teachers or parents). This is relatively straightforward and has the advantage of capturing crimes irrespective of whether they appear on any official records. However, measurement error may be introduced through recall bias (not being able to accurately recall past behaviours), or social desirability bias (choosing not to disclose certain behaviours). Further, there is a potential for measurement error based on questionnaire design (e.g. study wording or response options) and valuable data may not be recorded (e.g. details of criminal behaviour). Finally, a known limitation of cohort studies is that attrition is associated with socio-economic, demographic and health status which, in turn, may be associated with criminal behaviour. By relying on self-report measures of criminality, it is likely that cohort studies underestimate rates of criminality compared to the wider population ^4^.

Record linkage of cohort data to official police records has the potential to address some of the limitations of self-reported data. As official records are not affected by recall or social desirability bias, they can potentially provide greater detail and accuracy than would be feasible via self-report. Furthermore, attrition bias can be addressed using record linkage as participants’ criminality outcomes can be followed in cases where they miss opportunities to participate in study data collections. However, not all crimes come to the attention of the police or result in a formal record and so to rely solely on police records would under-estimate the prevalence of criminality in a cohort ^5^. There is evidence to suggest that violence between people who know each other, less serious violence and violence that involves alcohol are less likely to be reported and males are also less likely to report violent victimisation to the police than females ^5^. Furthermore, violence that involves injury or weapons and violence perpetrated by a stranger are more likely to come to the police’s attention. Finally, there is some evidence of a deprivation-related bias wherein offences against residents of the most deprived neighbourhoods are less likely to be reported to the police than offences against residents of less deprived areas ^6^. The impact of this on accurate estimates would be enhanced where the factors (e.g. ethnicity) associated with policing practice were also predictive of failure to participate in study follow-up. In police records, data quality issues within the records may also lead to error (e.g. failure to link resulting from poor or inaccurate personal identifiers) and that this may disproportionately impact some population groups.

In sum, a combination of official police records with self-reported criminal behaviours could allow research that uses the strengths of both sources of crime data by addressing some of their respective limitations. However, in the case of linking cohorts and police records, it is currently unclear whether the levels of criminality are sufficient for a longitudinal population study to be a viable resource for future research projects. Furthermore, gaining a better understanding of what age crimes are committed and in which areas can help to identify key age periods and geographical locations for where data linkage may be the most valuable for research.

As with all data linkage projects in longitudinal studies, there are specific considerations relating to data protection and confidentiality and wider considerations relating to participant trust and the acceptability of novel forms of data use. In the UK, criminal records were deemed ‘sensitive’ data in the Data Protection Act 1998 and are now considered ‘special category’ data in the EU General Data Protection Regulations (GDPR) and the UK’s Data Protection Act 2018 (DPA). Both categories are subject to elevated levels of protection. The DPA 1998 allowed for the use of criminal records where studies gained explicit consent from study participants or where the data was anonymised (and therefore no longer relatable to an individual, thus no longer being subject to data protection and confidentiality law). In contrast, the new DPA 2018 provides a separate legal basis for using identifiable ‘special category’ records for scientific research which is in the public interest, subject to utilising sufficient safeguards (GDPR Article 89). Nevertheless, these routes available to meet DPA 2018 requirements do not alter the requirement for research use of individual data to meet the Common Law Duty of Confidentiality, which can be met through consent, anonymisation or meeting a public interest test. However, data linkage based on consent may systematically omit some individuals and population sub-groups and introduce bias into study findings. Therefore, alternative mechanisms to use data for individuals who have not necessarily provided consent are needed to minimise the risk of selection bias. Further to addressing the legal basis for record linkages, it is also necessary to examine the acceptability of data linkage to crime records for cohort participants and – in order to justify the intrusion to privacy of non-consented approaches - to determine whether the group of participants who do consent to data linkage are, in terms of criminal behaviour, representative of the wider cohort (in which case consent could be a practical basis for this data use).

This paper describes a pilot linkage project of participants from the Avon Longitudinal Study of Parents and Children (ALSPAC) to criminal conviction and official caution records in the UK Police National Computer (PNC) database. This pilot aimed to test the feasibility of the linkage process. To our knowledge, this pilot project is the first to link criminal records to an English general population longitudinal cohort. This complements linkage to studies established within the criminal justice system (e.g. the Surveying Prisoner Crime Reduction study ^7^) or focused on criminality outcomes (e.g., the Edinburgh Study of Youth Transition and Crime ^8^) through linkage to Scottish criminality records. Outside of the UK, these linkages exist within Scandinavian studies using criminal registry records (e.g. the Swedish National Cohort Study ^9^).

The linkage in our pilot was restricted to an anonymous data extract of historic criminal convictions and cautions of ALSPAC study participants. No identifiers are present in the file meaning it cannot be linked to any participant records held within the ALSPAC databank. The aim of the project was to answer the following research questions: (1) What can participant responses to the study’s proposed linkage to criminality records suggest about the level of acceptability of this to ALSPAC participants? (2) Are there sufficient levels of recorded criminality for the data resource to be useful in future research? (3) During what age periods covered by the linkage are crimes most often committed? (4) In what geographical area that includes ALSPAC participants are crimes most commonly committed? (5) Are those we have consent to link to crime data representative of the wider cohort in terms of their self-reported criminal behaviours?

## Methods

### Avon Longitudinal Study of Parents and Children

ALSPAC is a birth cohort study that recruited pregnant women who were resident in and around the city of Bristol, with a due date between April 1991 and December 1992. Full details are available in the cohort profiles ^10, 11^ and a searchable data dictionary can be accessed from the study’s website ( http://www.bristol.ac.uk/alspac/researchers/access/). In brief, there were 14,541 pregnancies resulting in 13,988 children alive at one year of age. By age 18 years, an additional 713 children, who were eligible under the original study eligibility definition, but whose mothers had not joined the study during pregnancy, have also been recruited. This means there is a total of 15, 247 (75.3% of eligible) enrolled pregnancies and, from these pregnancies, there were 14,775 live-born children, of which 14,701 were alive at one year of age. The mothers, their partners, and the study children have been followed ever since through questionnaires and clinic visits.

### The Project to Enhance ALSPAC through Record Linkage (PEARL)

When the ALSPAC children reached legal adulthood (age 18 years), there was a postal campaign that aimed to re-enrol them into the study and to seek permission for linkage to their routine health and administrative records, including education, employment earnings and benefits, and criminal conviction and caution records. This was part of the Wellcome Trust funded ‘Project to Enhance ALSPAC through Record Linkage’ (PEARL). Each participant was sent a pack that included an information booklet and consent form, which provided a clear means to opt out of ALSPAC, or to any of the proposed linkages. Due to factors related to the negotiation of access to linked health records (i.e. unrelated to this crime data linkage), the participant information materials were initially issued in two batches. Batch one sought opt-in consent, while batch two was structured as an opt-out approach and notified participants that their routine records would be linked to ALSPAC in the event of non-response. Participants that did not respond to batch 1 were a sent a new opt-out pack. Following participant consultation, the opt-in/out materials were structured as a series of specific options to allow for individual level decision making. This led to participants returning forms that in effect provided consent for some linkage categories (e.g. an individual may have objected to the study’s use of their employment, earnings and benefits records while consenting to the study’s use of other records). The following participants were excluded from the pilot crime linkage: participants who no longer wished to be part of ALSPAC; those who objected to linkage to their criminal conviction and caution records; those where we had evidence the participant had not received their information pack (e.g. it was returned by the postal service as ‘addressee unknown’); and those who lacked capacity to consent. Due to the inclusion of a randomised controlled trial of linkage information materials ^12^ and other study factors, the participants selected to be in batch 1 and batch 2 were not selected at random and are likely to over represent participants with good histories of study participation.

Following the ALSPAC – MoJ pilot linkage, the study continued to issue opt-out linkage materials to all participants via postal campaigns and online promotion. Where practicable, consent was sought where participants attended a study data assessment visit. This means there is an increasing proportion of participants who have opted in to record linkage over time.

### Linkage of ALSPAC to Police National Computer (PNC) data

Following negotiations between ALSPAC and the Ministry of Justice (MoJ), it was agreed to conduct a *pilot* linkage exercise which would test the feasibility of the linkage mechanism through the production of an anonymous linked extract. For individuals for whom ALSPAC had permission to link to crime records (those who opted in to crime linkage from batch 1 or batch 2, and non-responders to batch 2 - except excluded cases), the following identifiers were sent to the MoJ: forename, surname, date of birth, current address, last four known addresses. This linkage was done in March 2013. The dataset provided by the MoJ contains no identifiers, meaning it cannot be linked to any other ALSPAC data.

Requests to link to the PNC are processed by a statistical research team within the UK Ministry of Justice (MoJ). ALSPAC securely provided the MoJ with the standardised personal identifiers of participants in the linkage sample for the sole purpose of conducting this linkage exercise. No attribute data about the participants was provided. The MoJ conducted the linkage to the PNC using a deterministic linkage protocol with manual review (see MoJ documentation below). Once linked, the MoJ provided an anonymised data extract detailing all historic criminal convictions and cautions that were linked to study participants. Direct individual identifiers were removed and replaced with two pseudonymised identifiers: 1) ‘lcr_id’, which uniquely identified individuals in the data set; and, 2) ‘lcr_caseid’, which identified unique cases and the criminal acts associated with it, which were nested within each individuals overall record (i.e. each individual with a link would have one or more criminal event records associated with at least one ‘case’). ALSPAC has no means to reverse these pseudonyms to the participants’ personal identifiers. The extract was securely sent to the PEARL team for analysis within the PEARL Data Safe Haven (at the University of Bristol).

### Linkage protocol

The linkage was conducted by MoJ staff. In summary, they received a file of identifers from ALSPAC and then processed (cleaned) these. They then searched the Home Office Police National Computer (HOPNC) live database. Where matches were found, the individual’s PNC ID was extracted and subsequently used to extract criminality outcomes.

The automated HOPNC database search process returns a set of results, indicating varying levels of matching success according to a set of deterministic match rules. Matches are graded from 01 to 24, and in general, the higher the number, the more suspect the match. The process accommodates the tendency for criminal convictions to be assigned to alias identities rather than true identities. Each match level may be sub-divided into A or B levels, where B also uses data contained in Alias and AliasDateOfBirth tables. ‘Suspect’ matches are manually matched against the HOPNC live database by MoJ staff in order to obtain either an accurate PNCID or a status of no match.

ALSPAC was not provided with information on match strength or as to whether suspect matches were manually reconciled, dropped or retained. This was due to the primary aim of the project being to demonstrate the feasibility of subsequent research and to test the workflow process (i.e. the aims did require the full linkage protocol to be implemented).

### Cleaning & standardisation

The cleaning process used aimed to standardise identifiers prior to matching:

- Adding centuries to the PNCID year portion
- Limiting Gender / Sex to 1st character of ‘Male’ / ‘Female’ / ‘Unknown’
- Supplying dummy date of birth where none provided. (29 Feb 2004 suggested)
- Splitting forenames into 3 columns; First forename, Second forename & Other forenames
- Removal of Hyphens, spaces, apostrophes, fullstops, commas from name elements
- Removing leading zeros from ‘Nibnum’ field if provided, which converts it to a CRONumber
- Correcting date formats
- Removing rows with insufficient mandatory fields

### Match rules

The MoJ linkage operator followed a linkage protocol including manual check rules and rules for dealing with duplicate entries. Where in doubt, the operator was instructed to not establish a link which, theoretically, increases the rate of false negative linkages but reduces the rate of false positive linkages. Where there was a high degree of missing data (less than three of forename, familyname or date of birth) then no link would be established. Where duplicates exist, and there is no conclusive evidence from other PNC information that they are a link, then none of the candidate entries are set to a match. The full HOPNC linkage protocol of the time is available from the authors on request.

### Ethical and data owner approvals

Ethical approval for ALSPAC was obtained from the ALSPAC Ethics and Law Committee (ALEC) and the Local Research Ethics Committees. The PEARL project received approval from ALEC and the Haydock NHS Research Ethics Committee (REF: 10/H1010/70) for the use of NHS records. Approval for the MoJ to link ALSPAC participants to their PNC records was granted by the PNC Information Access Panel (PIAP). When the study children reached legal adulthood (age 18), ALSPAC initiated a postal fair processing campaign to formally re-enrol the children into the study (prior to this parent-based consent was mandatory, although from age 9 children assented to data collection as well) and to simultaneously establish permissions for ALSPAC to link to their health and administrative records. All participants have been offered the right to opt-out (which is respected). This approach was developed with participant involvement.

### Measures

Data was cleaned, managed and analysed using STATA version 15 ^13^. 

***Police National Computer (PNC) data.*** The variables provided by the MoJ included: date of offence; offence class (1- violence against person, 2 – sexual offences, 3 – burglary, 4 – robbery, 5 – theft and handling stolen goods, 6 – fraud and forgery, 7 – criminal damage, 8 – drug offences, 9 – other indictable offence, 10 – indictable motoring offence, 11 – summary offences excluding motoring, 12 – summary motoring offences, 21 – offences outside England and Wales, 23 – breach offences); police force that processed the case; adjudication code (guilty, caution/warning/reprimand); disposal type (absolute discharge, conditional discharge, fine, community penalty, immediate custody, other).

***ALSPAC data.*** A variable was derived to summarise crime linkage consent status at the time of the pilot linkage: opted in to crime linkage; non-responder to batch 2; not sent to MoJ for crime linkage (this includes those who opted out of ALSPAC or to crime linkage, those who were non-responders to batch 1, and those who never received a PEARL pack). A more recent (September 2019) crime linkage consent status was also summarised in a similar way. Measures related to family socio-economic position were reported by the mother during her pregnancy with the study child: family occupational social class, defined as the higher of maternal and paternal social class and categorised as high (I-IIIN, professional, managerial, and non-manual skilled occupations) and low (IIIM-IV, manual skilled, semi-skilled and unskilled occupations); highest maternal education (university degree, A level, O level, vocational/none); housing tenure (owned/mortgaged, privately rented, council rented, other); and financial difficulties (quartiles of score with range 0–15, where the upper quartile (6+) is considered high). Child variables included sex and ethnicity (reported by the mother - White, non-White [no further disaggregation was possible due to small numbers]). Each child’s engagement with ALSPAC was measured by a derived variable which specified how many ALSPAC questionnaires they had completed by the age of 18 years. Antisocial and criminal behaviours were reported by the children at ages 14, 15.5, 17.5 and 18 years.

***Statistical analyses.*** We used descriptive statistics to summarise the number of convictions and cautions (overall and by crime linkage consent status), the year the offences were committed (as a proxy for age of the participants), and where they were committed (which constabulary). We then compared participants for whom we had permission to send their identifiers to the MoJ to participants for whom we did not have such permissions in terms of child sex and ethnicity, early life family socio-economic position, and child-reported anti-social and criminal behaviours. Finally, we considered these child-reported measures by current crime linkage consent status. 

## Results

### Acceptability of linkage to criminality records

At the time of the pilot linkage (March 2013), batch 1 (sent in 2011 to 7,790 participants) sought opt-in consent, while batch 2 (n=5,379, which included 4,708 non-responders to batch 1) gave participants the option to opt out of linkage. This resulted in permission to link to the crime records of 7,361 participants (comprised of 2,966 who opted in to crime linkage, and 4,395 who were non-responders to batch 2). Note that these figures represent a moment in time. As of the present day (September 2019), out of 13,239 participants who have now been sent an opt-out PEARL pack, or have been asked in person for their explicit consent (e.g. when attending an ALSPAC clinic), with regards crime linkage: 5,063 (38%) have opted in, 7,622 (58%) have not responded, and 554 (4%) have opted out or withdrawn consent. This opt-out rate is only slightly higher than that observed for education and health records (both 3%), and lower than that for earnings and benefits records (6%). Of those responding who opted out of crime linkage (n= 477), 52% (n=247) opted out of all linkages and the remaining group consented to linkage to at least one other register.

Overall, there were only small differences between those sent for linkage (n=7,361) and those not sent for linkage in terms of family socio-economic position. However, there were differences between those who opted in for data linkage and those who did not respond to linkage requests ( Table 1). Those who did opt in (during batch 1) were less likely to come from a socio-economically disadvantaged background (according to characteristics measured at the time of their birth), have fewer financial difficulties and have mothers with a higher education level compared to those who did not respond to consent requests using an opt-out request during batch 2.

**Table 1.** Child and family socio-economic characteristics overall and by crime linkage consent status.

		Overall N=14,701	Identifiers sent to MoJ for linkage?			
			No N=7521	Yes		
				Yes: overall N=7357	Yes: opt-in consent N=2963	Yes: non-response batch 2 N=4394
**Child characteristics**						
Sex	Male	51.1	53.2	48.9	40.0	54.8
Ethnicity		N=12071	N=6202	N=5869	N=2723	N=3146
	Non-White	5.0	4.9	5.2	4.1	6.1
**Early-life family SEP**		N=12406	N=6363	N=6043	N=2755	N=3288
Maternal education	Degree	12.9	11.7)	14.1	22.2	7.3
	None/vocational	30.0	29.0	31.2	17.2	42.9
						
Housing tenure		N=13016	N=6658	N=6358	N=2749	N=3609
	Owned/mortgaged	73.4	75.0	71.6	86.0	60.6
Occupational social class ^1^		N=11494	N=5927	N=5567	N=2651	N=2916
	High (I&II)	55.1	54.7	55.5	67.7	44.3
	Low (IV & V)	5.9	5.9	6.0	2.9	8.8
Financial difficulties ^2^		N=12077	N=6183	N=5894	N=2696	N=3198
	None	35.9	35.5	36.3	44.7	29.2
	High	20.0	20.5	19.5	13.0	24.9

NOTE: sample numbers vary according to data availability for each demographic measure. MoJ, Ministry of Justice; SEP, socio-economic position. ^1^Social class based on highest social class of mother and partner. ^2^High financial difficulties classified as upper quartile (over 6 in a scale of 0–20).

### Levels of police-recorded criminality in the ALSPAC cohort

Of those whose identifiers were sent to the MoJ for linkage (n=7,361), 885 (12%) were successfully linked to a criminal record. These participants had a conviction or caution for 4,000 separate offences, comprising 2,635 criminal convictions and 1,365 official cautions, warnings or reprimands. Of those linked, 394 (44.5%) had received at least one conviction and 84 (9.5%) had received 10 or more convictions.

Summary offences excluding motoring was the offence class with the greatest number of offences, followed by theft and handling of stolen goods, breach offences, drug offences, and violence against the person (Figure 2). Almost a third (31.6%) of offences related to serious (defined as class 1-5) crimes.

### When and where crimes were committed

The majority of the offences (n=3,454, 86%) were committed in the area covered by the Avon and Somerset constabulary ( Table 2). Neighbouring areas and London generally had higher numbers than areas further from the study catchment area. The earliest linked records were recorded in March 2002 (when participants would have been aged between 11 and 12 years). Of the years covered (up to 2013), offences were carried out most commonly in 2009 (n=629, 16%; Figure 3), when participants were approximately 18 years old.

**Table 2.** Number of offences by police force.

Police force which dealt with offence ^1^	Offences overall N=4000 n (%)	Convictions N=2635 n (%)	Cautions/ reprimands/warnings N=2635 n (%)
Avon and Somerset	3454 (86.4%)	2317 (87.9%)	1137 (83.3%)
Gloucestershire	61 (1.5%)	32 (1.2%)	29 (2.1%)
Dyfed-Powys	52 (1.3%)	35 (1.3%)	17 (1.3%)
Leicestershire	44 (1.1%)	30 (1.1%)	14 (1.0%)
Devon and Cornwall	43 (1.1%)	11 (0.4%)	32 (2.3%)
Wiltshire	40 (1.0%)	36 (1.4%)	<5
Metropolitan Police (London)	40 (1.0%)	27 (1.0%)	13 (1.0%)
Other	266 (6.7%)	147 (5.6%)	119 (8.7%)

^1^Police forces where ≥40 offences by ALSPAC participants had been recorded are listed individually in the table; the rest are combined in the ‘other’ category.

### Representativeness of sample included in data linkage

Participants who were in our linkage sample self-reported fewer criminal behaviours than those excluded from the linkage sample. Regarding present-day consent status, those who have explicitly opted in to data linkage generally self-report fewer criminal behaviours and have lower proportion of missing data than those who have not responded to the consent campaign ( Table 3 and Table 4).

**Table 3.** Self-reported anti-social and criminal activities in adolescence by crime linkage consent status.

Age & ALSPAC Questionnaire	Behaviour	Identifiers sent to MoJ for linkage?			
		No N (%)	Yes: overall N (%)	Yes: opt-in consent N (%)	Yes: non-response batch 2 N(%)
**14 years**					
(CCQ)	Theft	56 (15.8%)	1,084 (19.2)	669 (19.3)	415 (21.0)
	Hit, kicked or punched someone on purpose	130 (37.0)	2,201 (39.3)	1,353 (37.2)	848 (43.1)
	Deliberately damaged or destroyed property	21 (6.0)	340 (6.1)	185 (5.1)	155 (7.9)
	Arson	<5	80 (1.4)	55 (1.0)	45 (2.3)
	Rowdy or rude in public place	29 (8.3)	672 (12.0)	370 (10.2)	302 (15.4)
	Carried knife or weapon	15 (4.3)	276 (4.9)	144 (4.0)	132 (6.7)
**15.5 years**					
(TF3)	Theft	47 (18.4)	937 (18.7)	590 (16.8)	347 (23.3)
	Hit, kicked or punched someone on purpose	57 (22.3)	1,001 (20.0)	626 (17.8)	375 (25.3)
	Deliberately damaged or destroyed property	34 (13.3)	569 (11.4)	332 (9.4)	237 (16.0)
	Arson	38 (14.9)	755 (15.1)	481 (13.7)	274 (18.5)
	Rowdy or rude in public place	47 (18.4)	846 (18.9)	596 (16.9)	350 (23.4)
	Carried knife or weapon	22 (8.6)	395 (7.9)	220 (6.2)	175 (11.8)
**17.5 years**					
(TF4)	Theft	15 (7.4)	360 (9.6)	264 (9.0)	96 (11.1)
	Hit, kicked or punched someone on purpose	7 (3.5)	208 (5.6)	136 (4.7)	72 (8.4)
	Deliberately damaged or destroyed property	5 (2.5)	139 (3.7)	93 (3.2)	46 (5.4)
	Arson	<5	32 (0.9)	24 (0.8)	8 (0.9)
	Rowdy or rude in public place	15 (7.5)	341 (9.1)	245 (8.4)	96 (11.3)
	Carried knife or weapon	<5	71 (1.9)	51 (1.8)	20 (2.3)
**18 years**					
(CCT)	Theft	16 (7.4)	255 (8.2)	209 (8.3)	46 (8.1)
	Hit, kicked or punched someone on purpose	10 (4.7)	220 (7.1)	157 (6.2)	63 (11.1)
	Deliberately damaged or destroyed property	<5	131 (4.0)	88 (3.5)	33 (5.8)
	Carried knife or weapon	<5	40 (1.3)	25 (1.0)	15 (2.6)

MoJ, Ministry of Justice; ALSPAC Questionnaires CCQ, TF3, TF4, CCT for more information see http://www.bristol.ac.uk/alspac/researchers/our-data/questionnaires/child-completed-questionnaires/.

**Table 4.** Self-reported contact with police by crime linkage consent status at age 18 years.

Behaviour	Identifiers sent to MoJ for linkage?			
	No ^1^	Yes: overall	Yes: opt-in consent	Yes: non- response batch 2
In trouble with police	26 (13.3)	521 (14.1)	352 (12.3)	169 (20.4)
Picked up by police and taken home	5 (2.6)	91 (2.5)	47 (1.6)	44 (5.3)
Picked up by police and taken to police station	<5	71 (1.9)	42 (1.5)	29 (3.5)
Told off/told to move by police officer	33 (16.9)	565 (15.2)	390 (13.6)	175 (21.0)
Stopped and told to empty pockets or bag	15 (7.7)	324 (8.8)	217 (7.6)	107 (12.9)
Received official police caution	<5	118 (3.2)	69 (2.4)	49 (5.9)
Received official police charge	<5	34 (1.2)	22 (2.7)	<5
Was put on trial in court	<5	19 (0.5)	10 (0.35)	9 (1.1)
Received any ‘punishment’ (ASBO, young offenders, fine)	<5	31 (0.8)	16 (0.6)	15 (1.8)
Any criminal justice involvement (caution, conviction, trial)	<5	139 (3.8)	86 (3.0)	53 (6.4)

^1^Linkage was not carried out for participants who no longer wished to be part of ALSPAC, those who objected to linkage to their criminal conviction and caution records, those where we had evidence the participant had not received their information pack (e.g. it was returned by the postal service as ‘addressee unknown’), and those who lacked capacity to consent. MoJ, Ministry of Justice; ASBO, antisocial behaviour order.

Using self-reported antisocial and criminal activity we found that of those who did not dissent to the use of data linkage: 2,106 (14%) participants self-reported conducting at least one form of theft; 3,117 (21%) had kicked, hit or punched someone; and, 883 (6%) had had contact with the police. Compared to participants that did not dissent to data linkage or those who agreed to continue in ALSPAC but dissented to all data linkage options, the proportion of criminal activity was higher for the group that specifically dissented to crime data linkage: 53 (23%) participants self-reported conducting at least one form of theft, 92 (40%) had kicked, hit or punched someone and 12 (28%) had had contact with police.

## Discussion

We completed a pilot record linkage in 2013 which linked ALSPAC participants to their criminality records held in the Police National Computer database; this identified 4,000 criminality records relating to 885 participants from a sample of 7,361. The pilot was conditional on the extract being anonymous and not able to be linked to information on individual participants within the ALSPAC databank. Fewer than 400 participants explicitly opted out of linkage to criminality records prior to the pilot linkage. Criminal behaviour is a potentially sensitive area and so it was a positive finding that almost 900 participants with criminal record(s) enabled the linkage to happen through either explicit consent (in response to the opt-in request) or not objecting (in response to the opt-out fair processing campaign). Our finding that 4% of the sample explicitly opted out of linkage to crime data linkage supports the view that linkage to crime data is acceptable to the majority of study participants. Based on summary statistics, the small proportion of participants who dissented specifically to crime data linkage had engaged in a greater level of criminality compared to rest the of sample, which suggests consent status may vary according to criminal behaviour and by inference, that some participants consider their criminality record to be sensitive. However, a greater number of participants reporting criminality did provide explicit consent, which could infer that participants trust the study to use these records appropriately for research. Whilst this could benefit from further research (ideally using mixed methods designs), this could inform future study designs and governance frameworks, and the considerations of ethical review boards.

In the sample of participants with criminal records, 4,000 convictions and cautions were recorded, many related to serious crimes. If the linkage were repeated today, we would expect the number of criminal records to be substantially higher because (1) we now have permission to link to a larger sample (n=12,685 as of September 2019, 85% of eligible live births) and (2) there would now be more than 6 years of additional data. Therefore, we believe that there is a sufficient level of criminality in the ALSPAC sample for it to be a useful resource for crime-related research. However, it is likely that data from ALSPAC does not have sufficient rates of less commonly committed crimes for these to be studied individually.

While all participants in the pilot were informed about the linkage and had not objected, only a sub-set of these had provided explicit consent. While rates for consenting to data linkage were high, we found evidence suggesting different rates of criminal activity, and socio-demographic differences, according to consenting status (i.e. opting in, opting out or not responding to the consent request). Participants that explicitly consented to data linkage were more likely to be female, have higher socioeconomic status, lower levels of missing data and were less likely to self-report criminal behaviour. This pattern is similar to that found for general participation in study activities. Based on summary statistics ( Table 1), participants that explicitly opted out of data linkage had a higher prevalence of self-reported criminal behaviour compared to those who opted in or did not response to consent requests. This suggests that active cohort participants, indicated by actively consenting to linkage, may report lower levels of criminal behaviour and studies using this sample may therefore underestimate rates of criminal behaviour in the full (eligible) study population. As such, it is necessary to consider the potential for selection bias when using a sample that relies on explicit opt in consent status when designing linkage methodologies and considering the appropriateness of data sharing requests.

The majority of offences in the pilot linkage were committed in the Avon and Somerset Constabulary, an area with a similar geographical footprint to the ALSPAC recruitment area. This suggests that linkage to local police data, which contains more detail than that held in the national PNC, would capture most offences (at least to age 18). Working at a local level provides the opportunity to identify areas of research of local importance. However, as an individual’s criminal career continues, criminal activity may become less geographically clustered and centralised national criminality records may be of increasing value.

### Strengths and limitations

There were several strengths and limitations to be considered in our study. We benefitted from the wealth of data available for demographic measures, self-reported crime collected at multiple time-points and consent status according to explicit opt in, opt out and non-response to data linkage. This information allowed us to observe potential patterns of demographic characteristics and self-reported criminality according to consent status that could be used to inform future studies.

The primary limitation was that the pilot only produced a fully anonymous file, which cannot be linked to the wider ALSPAC dataset. This limits the potential for this evaluation. It is also important to consider the quality of the data that is being linked from both sources and their potential limitations for answering questions in this research area. For MoJ data, successful linkage depends on reliable and accurate record keeping and testimony of individuals where they may be motivation to falsify records (i.e. using a false identity). For ALSPAC records, the use of self-reported measures of criminal behaviours are vulnerable to social desirability bias, although the figures provided here illustrate that many participants are willing to report criminal and anti-social activity. Furthermore, drop out by participants can lead to attrition bias and out of date information for a proportion of the recruited participants.

The quality of data linkage also relies on the accuracy of records in both datasets. While ALSPAC’s administrative database is generally of good quality, it is likely to be out of date for some participants who are lost to follow-up. For the PNC data, the identifier database is known to have accuracy problems and includes pseudonyms, out of date information and duplicates. The linkage methodology used relied on deterministic matching that incorporates fuzzy parameters (i.e. where the requirement for all elements of the personal identifiers are relaxed in varying combinations). In matching data, ALSPAC was not provided with a match quality score (which generates an estimate of the likelihood that two records relate to the same individual), which is counter to expectations that linkage quality estimates are transparent and available to the analyst ^14^.

Our evaluation is complicated by the differing permissions approaches used between batch 1 and batch 2, and the fact that the sub-sample of participants included in the linkage exercise were not selected at random. These weaknesses are mitigated by the fact that this research is intended to demonstrate viability rather than provide accurate association or prevalence estimates. Also, given that the sub-sample disproportionately included participants with strong levels of engagement, it can be hypothesised that this has led to an underestimate of recorded criminality within the sample.

## Conclusions

Data linkage between the ALSPAC cohort and criminal data recorded by the MoJ can be used to reduce potential selection bias and measurement error that are key limitations when using self-reported crime data within longitudinal studies. We found significant demographic differences and rates of criminality according to consent status of participants (i.e. given consent, explicit opt out of study, non-response to consent requests), which suggest that methods of securing data must be considered carefully in future studies to reduce the risk of bias and excluding marginalised and vulnerable young people from the benefits of public research.

This study illustrates the feasibility of linkage but offers limited insight into criminal behaviours in the ALSPAC cohort. Without the ability to anonymously link criminal register data and ALSPAC cohort data on an individual level, it is not possible to realise the full benefits of record linkage.

While this pilot study does illustrate the feasibility of data linkage, without the use of anonymous linked data it provides limited insight into criminal behaviours in the ALSPAC cohort; it is not possible to realize the full benefits that record linkage without the ability to link this extract at an individual level to the ALSPAC databank. Advances in privacy preserving record linkage and ‘Trusted Research Environment’ secure research infrastructure and legislative changes (Digital Economy Act, DPA) may now enable linkage and the joint analysis of linked study-criminal record data under sufficiently controlled conditions to mitigate potential risks to confidentiality and help ensure that this form of data use is publicly acceptable. Individual-level linkage would enable direct comparisons between police-collected and self-reported criminal data, inform statistical strategies to account for missing data and allow investigation of research questions related to the causal pathways to criminal behaviours using the wealth of life-course information collected by ALSPAC or other longitudinal studies. Once linked, these studies can therefore provide invaluable evidence to inform public health approaches to tackling crime.

## Data availability

### Underlying data

ALSPAC data access, including linked PNC data, is through a system of managed open access. The steps below highlight how to apply for access to ALSPAC data.

- Please read the ALSPAC access policy which describes the process of accessing the data in detail, and outlines the costs associated with doing so.
- You may also find it useful to browse the fully searchable research proposals database, which lists all research projects that have been approved since April 2011 including those using linked data.

For enquiries for using linked data, please contact pearl@alspac.ac.uk.
