# The potential for linking cohort participants to official criminal records: a pilot study using the Avon Longitudinal Study of Parents and Children (ALSPAC)

**DOI:** 10.12688/wellcomeopenres.16328.2

**Published:** 2022-09-05

**Authors:** Andy Boyd, Alison Teyhan, Rosie P. Cornish, Jazz Croft, Richard Thomas, Iain Brennan, John Macleod

**Affiliations:** 1 School of Population Health Sciences, University of Bristol, Bristol, BS8 2BN, UK; 2 MRC Integrative Epidemiology Unit, University of Bristol, Bristol, BS8 2BN, UK; 3 Department of Criminology and Sociology, University of Hull, Hull, HU6 7RX, UK; 4 The National Institute for Health Research Applied Research Collaboration West (NIHR ARC West), University Hospitals Bristol and Weston NHS Foundation Trust, Bristol, UK

**Keywords:** Criminal conviction, official caution, Ministry of Justice, Police National Computer database, record linkage, birth cohort, ALSPAC

## Abstract

**Introduction**: Linking longitudinal cohort resources with police-recorded records of criminal activity has the potential to inform public health style approaches to policing, and may reduce potential sources of bias from self-reported criminal data collected by cohort studies. A pilot linkage of police records to the Avon Longitudinal Study of Parents and Children (ALSPAC) allows us to consider the acceptability of this linkage, its utility as a data resource, differences in self-reported crime according to consent status for data linkage, and the appropriate governance mechanism to support such a linkage.

**Methods**: We carried out a pilot study linking data from the ALSPAC birth cohort to Ministry of Justice (MoJ) records on criminal cautions and convictions. This pilot was conducted on a fully anonymous basis, meaning we cannot link the identified records to any participant or the wider information within the dataset. Using ALSPAC data, we used summary statistics to investigate differences in socio-economic background and self-reported criminal activity by consent status for crime linkage. We used MoJ records to identify the geographic and temporal concentration of criminality in the ALSPAC cohort.

**Results**: We found that the linkage appears acceptable to participants (4% of the sample opted out), levels of criminal caution and conviction are high enough to support research, and that the majority of crimes occurred in Avon & Somerset (the policing area local to ALSPAC). Those who did not respond to consent requests had higher levels of self-reported criminal behaviour compared to participants who provided explicit consent.

**Conclusions**: These findings suggest that data linkage in ALSPAC provides opportunities to study criminal behaviour and that linked individual-level records could provide robust research in the area. Our findings also suggest the potential for bias when only including participants who have explicitly consented to data linkage, highlighting the limitations of opt-in consent strategies.

## Abbreviations

ALSPAC, Avon Longitudinal Study of Parents and Children

MoJ, Ministry of Justice

PNC, Police National Computer

SEP, socio-economic position

## Introduction

Policing in the UK increasingly seeks to take a public health approach to tackling crime, where the focus is on proactive prevention, the tackling of upstream risk factors, and on populations rather than individuals ^1^. This approach is multi-disciplinary, takes a joint agency approach, and relies on ‘the skilled use and interpretation of data and the evidence base to ensure that interventions are designed, delivered and tailored to be as effective as possible’ ^1, 2^. This can now be seen in operation within some UK police forces – for example, within Thames Valley Police ^3^. Epidemiological analysis is an important approach to identify risk and protective factors for criminal and antisocial behaviours. Police records of criminality (e.g. convictions and cautions) do not contain data relating to an individual’s exposure to potential risk factors for perpetrating crime, whereas longitudinal birth cohort studies have a wealth of data on the lives of their participants, and often their families, peers, and wider contexts, across the life course. Therefore, linking police data with cohort studies has the potential to add considerable value to research on criminal behaviour.

Accurate measures of participants’ criminal behaviours are necessary for any such research to be valid. Some cohorts contain their own measures of criminality – these may be self-reported by the participants or by related individuals (e.g. teachers or parents). While this is a relatively straightforward way of measuring such behaviours, and has the advantage of capturing crimes irrespective of whether they appear on any official records, measurement error may be introduced through recall error (not being able to accurately recall past behaviours), or social desirability bias (choosing not to disclose certain behaviours). Further, there is a potential for measurement error based on questionnaire design (e.g. study wording or response options) and valuable data may not be recorded (e.g. details of criminal behaviour). Finally, a known limitation of cohort studies is that attrition is associated with socio-economic, demographic and health status which, in turn, may be associated with criminal behaviour. By relying on self-report measures of criminality, it is likely that cohort studies underestimate rates of criminality compared to the wider population ^4^.

Record linkage of cohort data to official police records has the potential to address some of the limitations of self-reported data. As official records are not affected by recall error or social desirability bias, they can potentially provide greater detail and accuracy than would be feasible via self-report. Furthermore, attrition can be addressed using record linkage as criminality outcomes can be followed in individuals even if they miss opportunities to participate in study data collections. However, not all crimes come to the attention of the police or result in a formal record and so to rely solely on police records would under-estimate the prevalence of criminality in a cohort ^5^. There is evidence to suggest that violence between people who know each other, less serious violence, and violence that involves alcohol are *less* likely to be reported to the police, and males are less likely to report violent victimisation than females ^5^. Conversely, violence that involves injury or weapons, and violence perpetrated by a stranger are *more* likely to come to the police’s attention. Finally, there is some evidence that offences against residents of the most deprived neighbourhoods are less likely to be reported to the police than offences against residents of less deprived areas ^6^. The impact of this on accurate estimates would be enhanced where the factors (e.g. ethnicity) associated with policing practice were also predictive of failure to participate in study follow-up. In police records, data quality issues within the records may also lead to error (e.g. failure to link resulting from poor or inaccurate personal identifiers) and this may disproportionately impact some population groups. Linking cohort and official records also enables research questions to be addressed that would not otherwise be possible e.g. investigating self-reported and official records measuring different constructs and analysing discrepancies in these data sources, and comparing outcomes of those who self-report criminality vs. those with officially recorded criminality (and thus, those who have come to the attention of the justice system) ^7, 8^.

In sum, a combination of official police records with self-reported criminal behaviours could allow research that uses the strengths of both sources of crime data while addressing some of their respective limitations. However, achieving linkage of a longitudinal cohort to any routine health or administrative data can be a complex and time-consuming process. In Scotland, the Edinburgh Study of Youth Transitions and Crime has successfully linked police records to a longitudinal population-based cohort ^7, 8^. That cohort study has a criminality focus, they work closely with Scottish criminal justice policy makers and practitioners, and police record linkage was integral to the study’s design from the start ^7, 8^. The legal, ethical and practical example set through their successful linkage therefore isn’t a precedent that other UK cohorts with a more general purpose can necessarily follow. Also, Scotland and England have differing legal systems and police records. However, linkages of police records to general cohorts have been achieved in other countries, such as the NSW-CDS cohort study in Australia ^9^, and the Swedish National Cohort Study ^10^.

As with all data linkage projects in longitudinal studies, there are specific considerations relating to data protection and confidentiality, and wider considerations relating to participant trust and the acceptability of novel forms of data use. In the UK, criminal records were deemed ‘sensitive’ data in the Data Protection Act 1998 and are now considered ‘special category’ data in the EU General Data Protection Regulations (GDPR) and the UK’s Data Protection Act 2018 (DPA). Both categories are subject to elevated levels of protection. The DPA 1998 allowed for the use of criminal records where studies gained explicit consent from study participants or where the data were anonymised (and therefore no longer relatable to an individual, thus no longer being subject to data protection and confidentiality law). In contrast, the new DPA 2018 provides a separate legal basis for using identifiable ‘special category’ records for scientific research which is in the public interest, subject to utilising sufficient safeguards (GDPR Article 89). Nevertheless, these routes available to meet DPA 2018 requirements do not alter the requirement for research use of individual data to meet the Common Law Duty of Confidentiality, which can be met through consent, anonymisation or meeting a public interest test. However, data linkage based on consent may systematically omit some individuals and population sub-groups and introduce bias into study findings. Therefore, alternative mechanisms to use data for individuals who have not necessarily provided consent are needed to minimise the risk of selection bias. Further to addressing the legal basis for record linkages, it is also necessary to examine the acceptability of data linkage to crime records for cohort participants and – in order to justify the intrusion to privacy of non-consented approaches - to determine whether the group of participants who do consent to data linkage are, in terms of criminal behaviour, representative of the wider cohort (in which case consent could be a practical basis for this data use).

It is also necessary to consider if any linkage is proportionate – to be ethical, it has to be useful. In the case of linking cohorts to police records, it is currently unclear whether the levels of criminality are sufficient for a longitudinal population study to be a viable resource for future research projects. Furthermore, gaining a better understanding of the age crimes are committed and in which areas can help to identify key age periods and geographical locations for where data linkage may be the most valuable for research.

This paper describes a pilot linkage project of participants from the Avon Longitudinal Study of Parents and Children (ALSPAC) to criminal conviction and official caution records in the UK Police National Computer (PNC) database held by the Ministry of Justice (MoJ). To our knowledge, this pilot project is the first to link criminal records to an English general population longitudinal cohort. The overall aim of this pilot was to test the feasibility of linking ALSPAC to official criminality records, and to determine if full linkage is likely to be worthwhile in terms of creating a useful resource for future research. Our specific research questions were: (1) What can participant responses to the study’s proposed linkage to criminality records suggest about the level of acceptability of this to ALSPAC participants? (2) Are there sufficient levels of recorded criminal caution or conviction for the data resource to be useful in future research? (3) In what geographical area are crimes most commonly committed by ALSPAC participants? (4) Are those we have consent to link to crime data representative of the wider cohort in terms of their self-reported criminal behaviours?

The linkage in our pilot was restricted to an anonymous data extract of historic criminal convictions and cautions of ALSPAC study participants. No identifiers are present in the file meaning it cannot be linked to any participant records held within the ALSPAC databank.

## Methods

### Avon Longitudinal Study of Parents and Children

ALSPAC is a birth cohort study that recruited pregnant women who were resident in and around the city of Bristol, with a due date between April 1991 and December 1992. Full details are available in the cohort profiles ^11, 12^ and a searchable data dictionary can be accessed from the study’s website ( http://www.bristol.ac.uk/alspac/researchers/access/). In brief, there were 14,541 pregnancies resulting in 13,988 children alive at one year of age (known as the ‘core sample’). By age 18 years, an additional 718 children, who were eligible under the original study eligibility definition, but whose mothers had not joined the study during pregnancy, had also been recruited. The mothers, their partners, and the study children have been followed ever since through questionnaires and clinic visits.

### The Project to Enhance ALSPAC through Record Linkage (PEARL)

When the ALSPAC children reached legal adulthood (age 18 years), there was a postal campaign that aimed to re-enrol them into the study and to seek permission for linkage to their routine health and administrative records, including education, employment, earnings and benefits, and criminal conviction and caution records (hereafter, ‘criminality records’). This was part of the Wellcome Trust funded ‘Project to Enhance ALSPAC through Record Linkage’ (PEARL). Each participant was sent a pack that included an information booklet and consent form, which provided a clear means to opt-out of ALSPAC, or to any of the proposed linkages. Due to factors related to establishing an appropriate ethico-legal basis for record linkage in ALSPAC and the negotiation of access to linked health records (i.e. unrelated to this crime data linkage), the participant information materials were initially issued in two batches. Batch one sought opt-in consent, which stated that linkage would only occur with explicit participant approval, while batch two was structured as an opt-out approach and notified participants that their routine records would be linked to ALSPAC unless they specifically opted-out (i.e. linkage would occur in the event of non-response). Participants that did not respond to batch 1 were a sent a new opt-out pack. Following participant consultation, the opt-in/out materials were structured as a series of specific linkage permission options to allow for individual level decision making. This led to participants returning forms that in effect indicated consent for some linkage categories even when the overall campaign was structured as an opt-out (e.g. an individual may have objected to the study’s use of their employment, earnings and benefits records while consenting to the study’s use of other records). The following participants were excluded from the pilot criminality linkage: participants who no longer wished to be part of ALSPAC; those who objected to linkage to their criminality records; those where we had evidence the participant had not received their information pack (e.g. it was returned by the postal service as ‘addressee unknown’); and those who lacked capacity to consent. Due to the inclusion of a randomised controlled trial of linkage information materials ^13^ and other study factors, the participants selected to be in batch 1 and batch 2 were not selected at random and are likely to over represent participants with good histories of study participation.

Following the ALSPAC – MoJ pilot linkage, the study continued to issue opt-out linkage materials to all participants via postal campaigns and online promotion. Where practicable, consent was sought where participants attended a study clinic visit. This means there is an increasing number of participants who have opted-in to record linkage over time.

### Linkage of ALSPAC to Police National Computer (PNC) data

The Police National Computer (PNC) is a large administrative database that was started in 1974 and contains information about police cautions and court convictions held on individual offenders in England and Wales ^14, 15^. Following negotiations between ALSPAC and the MoJ, it was agreed to conduct a *pilot* linkage exercise which would test the feasibility of the linkage mechanism through the production of an anonymous linked extract. For individuals for whom ALSPAC had permission to link to criminality records (those who opted-in to crime linkage from batch 1 or batch 2, and non-responders to batch 2 - except excluded cases), the following identifiers were sent to the MoJ: forename, surname, date of birth, current address, last four known addresses. No attribute data about the participants was provided. This linkage was done in March 2013.

The MoJ conducted the linkage to the PNC using a deterministic linkage protocol with manual review (see ’Linkage Protocol’ section below). Once linked, the MoJ provided an anonymised data extract detailing all historic criminal convictions and cautions that were linked to study participants. Direct individual identifiers were removed and replaced with two pseudonymised identifiers: 1) ‘lcr_id’, which uniquely identified individuals in the data set; and, 2) ‘lcr_caseid’, which identified unique cases and the criminal acts associated with it, which were nested within each individual’s overall record (i.e. each individual with a link would have one or more criminality event records associated with at least one ‘case’). ALSPAC has no means to reverse these pseudonyms to the participants’ personal identifiers. The extract was securely sent to the ALSPAC data linkage team for analysis within their PEARL Data Safe Haven (at the University of Bristol).

### Linkage protocol

The linkage was conducted by MoJ staff. In summary, they received a file of identifiers from ALSPAC and then processed (cleaned) these. They then searched the Home Office Police National Computer (HOPNC) live database. Where matches were found, the individual’s PNC ID was extracted and subsequently used to extract criminality outcomes.

The automated HOPNC database search process returns a set of results, indicating varying levels of matching success according to a set of deterministic match rules. Matches are graded from 01 to 24, and in general, the higher the number, the more suspect the match. The process accommodates the tendency for criminal convictions to be assigned to alias identities rather than true identities. Each match level may be sub-divided into A or B levels, where B also uses data contained in Alias and AliasDateOfBirth tables. ‘Suspect’ matches are manually matched against the HOPNC live database by MoJ staff in order to obtain either an accurate PNCID or a status of no match.

ALSPAC was not provided with information on match strength or as to whether suspect matches were manually reconciled, dropped or retained. This was due to the primary aim of the project being to demonstrate the feasibility of subsequent research and to test the workflow process (i.e. the aims did not require the full linkage protocol to be implemented).

### Cleaning & standardisation

The cleaning process used aimed to standardise identifiers prior to matching:

- Adding centuries to the PNCID year portion
- Limiting Gender / Sex to 1st character of ‘Male’ / ‘Female’ / ‘Unknown’
- Supplying dummy date of birth where none provided. (29 Feb 2004 suggested)
- Splitting forenames into 3 columns; First forename, Second forename & Other forenames
- Removal of hyphens, spaces, apostrophes, full stops, commas from name elements
- Removing leading zeros from ‘Nibnum’ field if provided, which converts it to a CRONumber
- Correcting date formats
- Removing rows with insufficient mandatory fields

### Match rules

The MoJ linkage operator followed a linkage protocol including manual check rules and rules for dealing with duplicate entries. Where in doubt, the operator was instructed to not establish a link which, theoretically, increases the rate of false negative linkages but reduces the rate of false positive linkages. Where there was a high degree of missing data (less than three of forename, familyname or date of birth) then no link would be established. Where duplicates exist, and there is no conclusive evidence from other PNC information that they are a link, then none of the candidate entries are set to a match. The full HOPNC linkage protocol of the time is available from the authors on request.

### Ethical and data owner approvals

Ethical approval for ALSPAC was obtained from the ALSPAC Ethics and Law Committee (ALEC) and the Local Research Ethics Committees. The PEARL project received approval from ALEC and the Haydock NHS Research Ethics Committee (REF: 10/H1010/70) for the use of NHS records. Approval for the MoJ to link ALSPAC participants to their PNC records was granted by the PNC Information Access Panel (PIAP). When the study children reached legal adulthood (age 18), ALSPAC initiated a postal fair processing campaign to formally re-enrol the children into the study (prior to this parent-based consent was mandatory, although from age 9 children assented to data collection as well) and to simultaneously establish permissions for ALSPAC to link to their health and administrative records. All participants have been offered the right to opt-out (which is respected). This approach was developed with participant involvement.

### Measures

Data was cleaned, managed and analysed using STATA version 15 ^16^.

***Police National Computer (PNC) data.*** The variables provided by the MoJ included: date of offence; offence class (1- violence against person, 2 – sexual offences, 3 – burglary, 4 – robbery, 5 – theft and handling stolen goods, 6 – fraud and forgery, 7 – criminal damage, 8 – drug offences, 9 – other indictable offence, 10 – indictable motoring offence, 11 – summary offences excluding motoring, 12 – summary motoring offences, 21 – offences outside England and Wales, 23 – breach offences); police force that processed the case; adjudication code (guilty, caution/warning/reprimand); disposal type (absolute discharge, conditional discharge, fine, community penalty, immediate custody, other).

***ALSPAC data.*** A variable was derived to summarise criminality linkage consent status at the time of the pilot linkage: opted-in to criminality linkage; non-responder to batch 2; not sent to MoJ for criminality linkage (this includes those who opted-out of ALSPAC or to criminality linkage, those who were non-responders to batch 1, and those who never received a PEARL pack). Current (September 2019) criminality linkage consent status was also summarised in a similar way. Measures related to family socio-economic position (SEP) were reported by the mother during her pregnancy with the study child: family occupational social class, defined as the higher of maternal and paternal social class and categorised as high (I-IIIN, professional, managerial, and non-manual skilled occupations) and low (IIIM-IV, manual skilled, semi-skilled and unskilled occupations); highest maternal education (university degree, A level, O level, vocational/none); housing tenure (owned/mortgaged, privately rented, council rented, other); and financial difficulties (quartiles of score with range 0–15, where the upper quartile (6+) is considered high). Child variables included sex and ethnicity (reported by the mother - White, non-White [no further disaggregation was possible due to small numbers]).

Antisocial and criminal behaviours were reported by the children at ages 14, 15.5, 17.5 and 18 years. A series of binary variables were derived based on whether they reported doing each of the behaviours in the previous 12 months (no, yes): theft (includes stealing cars, from a person, from a shop etc.); hit, kicked or punched someone on purpose; carried a knife or weapon for protection or use during a fight; deliberately damaged or destroyed property belonging to someone else; deliberately set fire to property or building (or attempted to); rowdy or rude in a public place. At 17.5 years, they also reported a series of measures related to having had involvement with the police and criminal justice system in the past year. A series of binary variables were derived (no/yes): in trouble with police; picked up by police and taken home; picked up by police and taken to station; told off/told to move on by police officer; stopped and told to empty pockets or bag; received official police caution; charged for committing a crime; been on trial in court. Due to the small numbers reporting these outcomes, two further aggregate variables were derived: received any ‘punishment’ (answered yes to receiving fine, in a young offenders’ institution, having an Antisocial Behaviour Order (ASBO), or having mediation as an offender); and any criminal justice involvement (answered yes to having had a caution, conviction, being on trial, or receiving a ‘punishment’).

***Statistical analyses.*** We used descriptive statistics to summarise the number of convictions and cautions, the year the offences were committed (as a proxy for age of the participants), and where they were committed (which policing area). We then used ALSPAC questionnaire date to compare: (1) participants whose identifiers were sent to the MoJ to those whose identifiers were not sent; and (2) within the sent for linkage group, the participants who explicitly opted-in to linkage to those in batch 2 who did not opt-out, in terms of child sex and ethnicity, early life family SEP, and child-reported anti-social and criminal behaviours. Finally, we repeated these comparisons by current criminality linkage consent status. For these descriptive analyses, we excluded triplets and quadruplets (as their ALSPAC data are not released to researchers) and those who have withdrawn consent from ALSPAC participation, giving a sample size of 14,683. Note that due to missing data in the ALSPAC measures, the denominator for each individual comparison varies.

## Results

### Acceptability of linkage to criminality records

**Figure 1.**
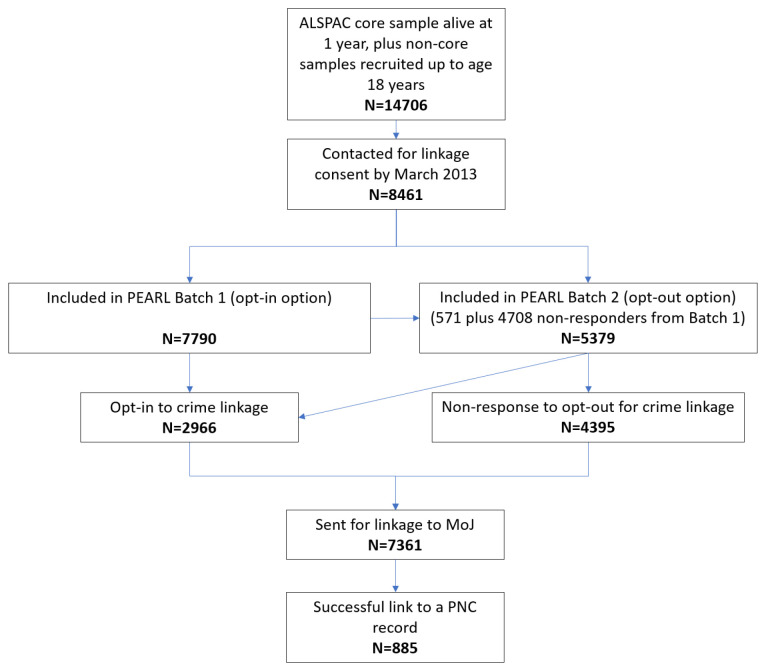
Flow chart of ALSPAC participants included in the pilot linkage to MoJ crime data.

At the time of the pilot linkage (March 2013), batch 1 (sent in 2011 to 7,790 participants) sought opt-in consent, while batch 2 (n=5,379, which included 4,708 non-responders from batch 1) gave participants the option to opt-out of the linkage, which would proceed in the event of non-response (given the granular nature of the opt-out form, some respondents to batch 2 opt-in to criminality linkages while opting-out of other linkage data sources). This resulted in permission to link to the criminality records of 7,361 participants (comprised of 2,966 who opted-in to crime linkage, and 4,395 who were non-responders to batch 2) ( Figure 1). Note that these figures represent a moment in time. Since the pilot linkage, there has been an increase in the number of participants for whom we have permission to link to criminality records. As of the present day (September 2019), out of 13,239 participants who have now been sent an opt-out PEARL pack, or have been asked in person for their explicit consent at a point where this was practicable (e.g. when attending an ALSPAC clinic), with regards criminality linkage: 5,062 (38%) have opted-in, 7,619 (58%) have not responded, 477 (4%) have opted-out of criminality linkage, and 81 (<1%) have withdrawn from the ALSPAC study overall. The criminality opt-out rate is only slightly higher than that observed for education and health records (both 3%), and lower than that for earnings and benefits records (6%). Of the 477 who have opted-out of criminality linkage, 52% (n=247) have opted-out of all linkages, while the rest have consented to linkage to at least one other linkage data source. Note that the current day numbers include 177 participants (174 opt-in and 3 non-response) who enrolled with ALSPAC after the pilot linkage: these participants are not included in the analysis by current day consent status below as they do not have questionnaire data at earlier timepoints.

### Levels of police-recorded criminality in the ALSPAC cohort

Of those whose identifiers were sent to the MoJ for linkage (n=7,361), 885 (12%) were successfully linked to a criminality record. These participants had a conviction, caution, reprimand or warning for 4,000 separate offences, comprising 2,635 criminal convictions and 1,365 official cautions, warnings or reprimands. Of those linked, 394 (44.5%) had received at least one conviction and 84 (9.5%) had received 10 or more convictions.

The offence class with the greatest number of offences was summary offences excluding motoring, followed by theft and handling of stolen goods, breach offences, drug offences, and violence against the person. Almost a third (31.6%) of offences related to serious crimes (defined as class 1-5).

### Where and when crimes were committed

**Figure 2.**
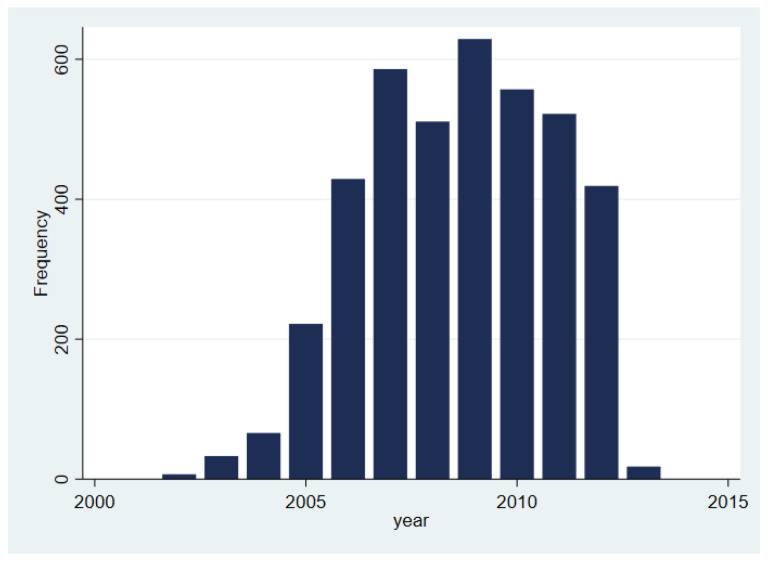
Ministry of Justice recorded convictions and cautions according to estimated start year of perpetration.

The majority of the offences (n=3,454, 86%) were committed in the area covered by the Avon and Somerset constabulary ( Table 1). Neighbouring areas and London generally had higher numbers than areas further from the study catchment area. The earliest linked records were recorded in March 2002 (when participants would have been aged between 11 and 12 years). Of the years covered (up to 2013), offences were carried out most commonly in 2009 (n=629, 16%; Figure 2), when participants were approximately 18 years old.

**Table 1.** Number of offences by police force.

Police force that dealt with offence ^1^	Offences overall N=4000 n (%)	Convictions N=2635 n (%)	Cautions/reprimands/ warnings N=1365 n (%)
Avon and Somerset	3454 (86.4%)	2317 (87.9%)	1137 (83.3%)
Gloucestershire	61 (1.5%)	32 (1.2%)	29 (2.1%)
Dyfed-Powys	52 (1.3%)	35 (1.3%)	17 (1.3%)
Leicestershire	44 (1.1%)	30 (1.1%)	14 (1.0%)
Devon and Cornwall	43 (1.1%)	11 (0.4%)	32 (2.3%)
Wiltshire	40 (1.0%)	36 (1.4%)	4 (0.3%)
Metropolitan Police (London)	40 (1.0%)	27 (1.0%)	13 (1.0%)
Other	266 (6.7%)	147 (5.6%)	119 (8.7%)

^1^Police forces in England & Wales where ≥40 offences by ALSPAC participants had been recorded are listed individually in the table; the rest are combined in the ‘other’ category.

### Representativeness of sample included in data linkage

Of the 14,683 participants included in our analyses, 7357 (50.1%) had their identifiers sent to the MoJ for linkage (2963 of these had opted-in and 4394 were Batch 2 non-responders) and 7326 did not have their identifiers sent (this group includes those who opted-out of linkage to criminality records, and those who had not received a consent pack – either because they had not been included in the consent campaign by March 2013, or their pack was returned to sender). The overall pattern was of greater differences *within* the sent for linkage group (i.e. between those who were opt-in and those who were non-responders) than between the sent for linkage group overall compared to the not sent for linkage group. Those who opted-in to criminality data linkage were more likely to be female, of White ethnicity, and from a socio-economically advantaged background, compared to those in batch 2 who did not respond to the opt-out request ( Table 2). Participants who were in the linkage sample generally reported fewer criminal behaviours than those excluded from the linkage sample ( Table 3). Further, those who opted-in generally reported fewer criminal behaviours than those who were non-responders. The pattern was broadly similar for contact with the criminal justice system, although the proportion of participants reporting such outcomes was small ( Table 4).

**Table 2.** Child and family socio-economic characteristics overall and by crime linkage consent status at time of pilot linkage.

		Overall N=14,683	Identifiers sent to MoJ for pilot linkage in March 2013?			
			No N=7326	Yes: overall N=7357	Yes: opt-in consent N=2963	Yes: non- response (batch 2) N=4394
**Child characteristics**						
Sex	Male	51.3 (50.5-52.1)	53.8 (52.6-54.9)	48.9 (47.7-50.0)	40.0 (38.3-41.8)	54.8 (53.4-56.3)
Ethnicity		N=12071	N=6202	N=5869	N=2723	N=3146
	Non-White	5.0 (4.7-5.5)	4.9 (4.4-5.5)	5.2 (4.6-5.8)	4.1 (3.4-4.9)	6.1 (5.3-7.0)
**Early-life family SEP**		N=12406	N=6363	N=6043	N=2755	N=3288
Maternal education	Degree	12.9 (12.3-13.5)	11.7 (11.0-12.6)	14.1 (13.2-15.0)	22.2 (20.7-23.8)	7.3 (6.5-8.3)
	None/vocational	30.0 (29.2-30.9)	29.0 (27.9-30.1)	31.2 (30.0-32.3)	17.2 (15.8-18.7)	42.9 (41.2-44.6)
Housing tenure		N=13016	N=6658	N=6358	N=2749	N=3609
	Owned/mortgaged	73.4 (72.6-74.1)	75.0 (74.0-76.0)	71.6 (70.5-72.7)	86.0 (84.7-87.3)	60.6 (59.0-62.2)
Occupational social class		N=11494	N=5927	N=5567	N=2651	N=2916
	High (I&II)	55.1 (54.2-56.0)	54.7 (53.5-56.0)	55.5 (54.2-56.8)	67.7 (65.9-69.5)	44.3 (42.5-46.2)
	Low (IV & V)	5.9 (5.5-6.4)	5.9 (5.3-6.5)	6.0 (5.4-6.7)	2.9 (2.4-3.7)	8.8 (7.8-9.9)
Financial difficulties		N=12077	N=6183	N=5894	N=2696	N=3198
	None	35.9 (35.0-36.7)	35.5 (34.3-36.7)	36.3 (35.1-37.5)	44.7 (42.8-46.6)	29.2 (27.6-30.8)
	High	20.0 (19.3-20.7)	20.5 (19.5-21.5)	19.5 (18.5-20.5)	13.0 (11.8-14.4)	24.9 (23.4-26.4)

NOTE: sample numbers vary for each demographic measure due to missing data (with the exception of sex, which is complete). Numbers in table represent percentages and 95% confidence intervals.

**Table 3.** Self–reported anti–social and criminal activities in adolescence by crime linkage consent status at time of pilot linkage.

Age	N	Behaviour in past year	Identifiers sent to MoJ for linkage in March 2013?			
			No	Yes: overall	Yes: opt-in consent	Yes: non-response batch 2
**14 years**			% (95% CI) reporting each behaviour **within** each column ^1^			
	6170	Theft	19.2 (17.9-20.6)	18.4 (17.0-19.8)	16.9 (15.5-18.5)	25.7 (21.9-29.8)
	6121	Hit, kicked or punched someone on purpose	40.6 (38.9-42.3)	37.9(36.2-39.8)	36.0 (34.1-38.0)	47.8 (43.2-52.3)
	6089	Deliberately damaged or destroyed property	6.9 (6.1-7.9)	5.1 (6.1-7.9)	4.4 (3.6-5.3)	8.6 (6.4-11.5)
	6103	Arson	1.6 (1.2-2.1)	1.2 (0.8-1.6)	0.9 (0.6-1.4)	2.6 (1.5-4.5)
	6110	Rowdy or rude in public place	12.9 (11.8-14.1)	10.5 (9.5-11.7)	9.0 (7.9-10.2)	18.7 (15.4-22.5)
	6135	Carried knife or weapon	5.5 (4.8-6.4)	4.2 (3.5-5.0)	3.6 (2.9-4.4)	7.1 (5.1-9.8)
**15.5 years**						
	5368	Theft	21.7 (20.3-23.3)	15.5 (14.1-17.0)	14.9 (13.5-16.5)	21.2 (16.4-27.0)
	5361	Hit, kicked or punched someone on purpose	23.3 (21.7-24.8)	16.6 (15.2-18.2)	16.0 (14.5-17.6)	22.9 (17.9-28.8)
	5361	Deliberately damaged or destroyed property	14.0 (12.8-15.4)	8.7 (7.7-9.9)	8.2 (7.1-9.4)	13.9 (9.9-19.0)
	5361	Arson	17.1 (15.8-18.5)	12.9 (11.7-14.3)	12.7 (11.4-14.2)	15.2 (11.1-20.4)
	5360	Rowdy or rude in public place	21.9 (20.5-23.5)	15.6 (14.2-17.1)	14.9 (13.5-16.4)	22.6 (17.6-28.5)
	5363	Carried knife or weapon	10.3 (9.2-11.4)	5.6 (4.8-6.6)	5.3 (4.5-6.3)	8.7 (5.7-13.1)
**17.5 years**						
	4033	Theft	10.8 (9.5-12.3)	8.2 (7.1-9.4)	7.5 (6.4-8.7)	13.7 (10.0-18.5)
	4034	Hit, kicked or punched someone on purpose	6.7 (5.6-7.9)	4.2 (3.5-5.2)	3.9 (3.1-4.9)	6.7 (4.2-10.5)
	4027	Deliberately damaged or destroyed property	4.6 (3.7-5.6)	2.8 (2.2-3.6)	2.6 (2.0-3.4)	3.9 (2.1-7.2)
	4017	Arson ^2^	0.9 (0.5-1.4)	0.8 (0.5-1.3)	-	n<5
	4012	Rowdy or rude in public place	10.3 (9.0-11.8)	7.9 (6.8-9.1)	7.5 (6.4-8.8)	10.6 (7.4-15.1)
	4014	Carried knife or weapon	2.2 (1.6-3.0)	1.5 (1.1-2.1)	1.4 (1.0-2.1)	2.4 (1.1-5.2)
**18 years**						
	3347	Theft	8.8 (7.4-10.5)	4.2 (3.5-5.2)	3.9 (3.1-4.9)	6.7 (4.2-10.5)
	3346	Hit, kicked or punched someone on purpose	8.0 (6.6-9.6)	6.4 (5.4-7.6)	5.8 (4.9-7.0)	12.6 (8.4-18.5)
	3344	Deliberately damaged or destroyed property	3.9 (3.0-5.1)	3.6 (2.8-4.5)	3.4 (2.7-4.4)	5.2 (2.7-9.7)
	3346	Rowdy or rude in public place	10.3 (9.0-11.8)	7.9 (6.8-9.1)	7.5 (6.4-8.8)	10.6 (7.4-15.1)
	3347	Carried knife or weapon ^2^	1.6 (1.1-2.5)	1.1 (0.7-1.6)	-	n<5

^1^The N in each column differs by measure and time–point and is not shown in this table ^2^Numbers are suppressed in the ‘opt–in’ column for these variables to prevent calculation of the small n in the ‘non–response’ column

**Table 4.** Self–reported contact with criminal justice system by age 18 years by crime linkage consent status at time of pilot linkage.

Contact with criminal justice system in past year, reported at age 17.5 years	N	Identifiers sent to MoJ for linkage in March 2013?			
		No	Yes: overall	Yes: opt-in consent	Yes: non-response batch 2
		% (95% CI) reporting criminal justice system contact **within** each column ^1^			
In trouble with police	3940	18.0 (16.3-19.9)	10.9 (9.6-12.3)	10.5 (9.1-11.9)	14.2 (10.4-19.2)
Picked up by police and taken home	3947	3.4 (2.6-4.3)	1.8 (1.3-2.4)	1.3 (0.9-2.0)	5.2 (3.1-8.8)
Picked up by police and taken to police station	3944	2.4 (1.8-3.2)	1.5 (1.1-2.1)	1.4 (1.0-2.1)	2.0 (0.8-4.8)
Told off/told to move on by police officer	3951	17.9 (16.2-19.7)	13.3 (11.9-14.8)	12.3 (10.9-13.9)	20.6 (16.0-26.2)
Stopped and told to empty pockets or bag	3949	10.2 (8.9-11.7)	7.4 (6.4-8.6)	7.0 (5.9-8.2)	10.5 (7.3-15.0)
Received official police caution	3934	4.1 (3.3-5.1)	2.2 (1.7-2.9)	1.8 (1.3-2.5)	5.3 (3.1-9.0)
Received official police charge ^2^	3943	2.2 (1.6-2.9)	1.1 (0.7-1.6)	-	n<5
Was put on trial in court ^2^	3934	0.7 (0.4-1.2)	0.3 (0.2-0.7)	-	n<5
Has received any ‘punishment’ from criminal justice system (e.g. fine, ASBO, young offenders, community service, mediation process as offender) ^2^	3913	1.3 (0.9-2.0)	0.6 (0.4-1.1)	-	n<5
Any criminal justice involvement reported (reported yes to caution, conviction, trial, or ‘punishment’)	3918	4.9 (4.0-6.0)	2.8 (2.2-3.6)	2.4 (1.8-3.2)	6.3 (3.8-10.1)

ASBO, antisocial behaviour order ^1^The N in each column differs by measure and time–point and is not shown in this table ^2^Numbers are suppressed in the ‘opt–in’ column for these variables to prevent calculation of the small n in the ‘non–response’ column

The comparisons by current consent status included 4884 opt-in, 7612 non-responders, and 2187 individuals with no permission for linkage. Overall, the patterns observed by current day consent status in SEP ( Table 5), self-reported criminal behaviours ( Table 6), and contact with the criminal justice system ( Table 7) were similar to those observed by consent status at the time of the pilot linkage.

**Table 5.** Child and family socio-economic characteristics overall and by current crime linkage consent status.

		Consented to crime record linkage by September 2019?			
		No N=2187	Yes: overall N=12496	Yes: opt-in consent N=4884	Yes: non-response (batch 2) N=7612
**Child characteristics**					
Sex	Male	55.4 (53.3-57.4)	50.6 (49.7-51.5)	39.9 (38.6-41.3)	57.4 (56.3-58.5)
Ethnicity		N=1648	N=10423	N=4426	N=5997
	Non-White	5.1 (4.1-6.3)	5.0 (4.6-5.5)	4.0 (3.4-4.6)	5.8 (5.3-6.5)
**Early-life family SEP**		N=1714	N=10692	N=4483	N=6209
Maternal education	Degree	10.2 (8.9-11.7)	14.1 (13.2-15.0)	20.2 (19.1-21.4)	8.3 (7.7-9.1)
	None/vocational	34.0 (31.7-36.2)	31.2 (30.0-32.3)	18.7 (17.6-19.9)	37.1 (35.9-38.3)
Housing tenure		N=1914	N=11102	N=4489	N=6613
	Owned/mortgaged	68.7 (66.5-70.7)	74.2 (73.3-75.0)	84.3 (83.2-85.4)	67.3 (66.1-68.4)
Occupational social class		N=1560	N=9934	N=4296	N=5638
	High (I&II)	53.6 (51.1-56.1)	55.3 (54.3-56.3)	64.9 (63.8-66.3)	48.1 (46.8-49.4)
	Low (IV & V)	6.6 (5.5-7.9)	5.8 (5.4-6.3)	3.5 (3.0-4.1)	7.6 (6.9-8.3)
Financial difficulties		N=1666	N=10411	N=4376	N=6035
	None	34.2 (32.0-36.5)	36.1 (35.2-37.1)	42.8 (41.3-44.2)	31.3 (30.2-32.5)
	High	22.3 (20.3-24.3)	19.6 (18.9-20.4)	14.6 (13.6-15.7)	23.3 (22.2-24.4)

NOTE: sample numbers vary for each demographic measure due to missing data (with the exception of sex, which is complete). Numbers in table represent percentages and 95% confidence intervals.

**Table 6.** Self-reported anti-social and criminal activities in adolescence by current crime linkage consent status.

Age	Behaviour in past year	Consented to crime record linkage by September 2019?			
		No	Yes: overall	Yes: opt-in consent	Yes: non-response batch 2
**14 years**		% (95% CI) reporting each behaviour **within** each column ^1^			
	Theft	15.3 (17.9-20.6)	19.2 (18.2-20.2)	18.2 (17.0-19.5)	20.9 (19.1-22.7)
	Hit, kicked or punched someone on purpose	40.8 (36.6-45.1)	39.2(38.0-40.5)	37.2 (35.6-38.8)	43.0 (40.9-45.2)
	Deliberately damaged or destroyed property	6.1 (4.3-8.5)	6.1 (5.5-6.7)	5.1 (4.4-5.8)	7.9 (6.8-9.2)
	Arson	1.0 (0.4-2.3)	1.4 (1.1-1.8)	0.9 (0.7-1.3)	2.3 (1.7-3.1)
	Rowdy or rude in public place	10.1 (7.8-13.1)	12.0 (11.1-12.8)	10.1 (9.2-11.2)	15.4 (13.8-17.0)
	Carried knife or weapon	4.8 (3.3-7.0)	4.9 (4.4-5.5)	3.9 (3.4-4.6)	6.7 (5.7-7.9)
**15.5 years**					
	Theft	21.6 (17.6-26.2)	18.7 (17.6-19.8)	16.7 (15.5-18.0)	23.3 (21.2-25.5)
	Hit, kicked or punched someone on purpose	23.0 (18.9-27.7)	20.0 (18.9-21.1)	17.8 (16.5-19.0)	25.3 (23.1-27.5)
	Deliberately damaged or destroyed property	14.6 (11.3-18.7)	11.3 (10.5-12.3)	9.4 (8.5-10.4)	16.0 (14.2-17.9)
	Arson	16.9 (13.3-21.1)	15.1 (14.1-16.1)	13.6 (12.5-14.8)	18.5 (16.6-20.5)
	Rowdy or rude in public place	20.5 (16.6-25.0)	18.9 (17.8-20.0)	16.9 (15.7-18.2)	23.6 (21.5-25.9)
	Carried knife or weapon	11.2 (8.3-15.0)	7.9 (7.2-8.7)	6.2 (5.5-7.1)	11.8 (10.3-13.5)
**17.5 years**					
	Theft	8.0 (5.2-12.1)	9.5 (8.6-10.5)	9.0 (8.1-10.1)	11.1 (9.2-13.4)
	Hit, kicked or punched someone on purpose	3.2 (1.6-6.4)	5.5 (4.8-6.3)	4.7 (4.0-5.5)	8.4 (6.7-10.5)
	Deliberately damaged or destroyed property	2.4 (1.1-5.3)	3.7 (3.1-4.3)	3.2 (2.6-3.9)	5.3 (4.0-7.1)
	Arson ^2^	n<5	0.8 (0.6-1.2)	0.8 (0.6-1.2)	0.9 (0.5-1.9)
	Rowdy or rude in public place	8.1 (5.3-12.2)	9.1 (8.2-10.0)	8.4 (7.5-9.5)	11.3 (9.3-13.6)
	Carried knife or weapon	n<5	1.9 (1.5-2.4)	1.8 (1.3-2.3)	2.3 (1.5-3.6)
**18 years**					
	Theft	7.0 (4.4-11.0)	8.2 (7.3-9.2)	8.2 (7.2-9.3)	8.1 (6.1-10.6)
	Hit, kicked or punched someone on purpose	6.2 (3.7-10.0)	7.1 (6.2-8.0)	6.2 (5.3-7.2)	11.1 (8.7-13.9)
	Deliberately damaged or destroyed property	n<5	3.9 (3.2-4.6)	3.4 (2.8-4.2)	5.8 (4.1-8.0)
	Rowdy or rude in public place	8.1 (5.3-12.2)	9.1 (8.2-10.0)	8.4 (7.5-9.5)	11.3 (9.3-13.6)
	Carried knife or weapon	n<5	1.3 (0.9-1.8)	1.0 (0.7-1.5)	2.6 (1.6-4.3)

^1^The N in each column differs by measure and time-point and is not shown in this table

**Table 7.** Self-reported contact with criminal justice system by age 18 years by current crime linkage consent status.

Contact with criminal justice system in past year, reported at age 17.5 years	Consented to crime record linkage by September 2019?			
	No	Yes: overall	Yes: opt-in consent	Yes: non- response batch 2
	% (95% CI) reporting criminal justice system contact **within** each column ^1^			
In trouble with police	15.4 (11.3-20.5)	14.1 (13.0-15.2)	12.3 (11.1-13.5)	20.4 (17.8-23.3)
Picked up by police and taken home	3.3 (1.7-6.5)	2.5 (2.0-3.0)	1.6 (1.2-2.2)	5.3 (4.0-7.0)
Picked up by police and taken to police station	2.1 (0.9-4.9)	1.9 (1.5-2.4)	1.5 (1.1-2.0)	3.5 (2.4-5.0)
Told off/told to move on by police officer	15.4 (14.3-16.6)	18.2 (13.8-23.6)	15.2 (14.1-16.4)	21.0 (18.4-23.9)
Stopped and told to empty pockets or bag	7.9 (5.1-12.0)	8.7 (7.9-9.7)	7.5 (6.6-8.6)	12.9 (10.8-15.3)
Received official police caution	n<5	3.2 (2.7-3.8)	2.4 (1.9-3.0)	5.9 (4.5-7.8)
Received official police charge	2.5 (1.1-5.4)	1.5 (1.2-2.0)	1.2 (0.8-1.7)	2.7 (1.8-4.0)
Was put on trial in court	n<5	0.5 (0.3-0.8)	0.3 (0.2-0.6)	1.1 (0.6-2.1)
Has received any ‘punishment’ from criminal justice system (e.g. fine, ASBO, young offenders, community service, mediation process as offender)	n<5	1.0 (0.7-1.3)	0.7 (0.5-1.1)	1.8 (1.1-3.0)
Any criminal justice involvement reported (reported yes to caution, conviction, trial, or ‘punishment’)	2.9 (1.4-6.0)	3.8 (3.3-4.5)	3.1 (2.5-3.8)	6.5 (5.0-8.4)

ASBO, antisocial behaviour order ^1^The N in each column differs by measure and time-point and is not shown in this table

It is important to note that there are also differences in the proportions of missing data by consent status for each variable: those with opt-in consent have a lower proportion of missing data than those who have not responded to the consent campaign. This is true of both early-life (reported by participant’s mother) variables and those reported by the participant themselves later in adolescence. For example, of those who had opted-in at the time of the pilot, 7% are missing maternal education data, compared to 25% of those who were non-responders and 13% of those who were not sent for linkage (percentages by current day consent status are 8%, 18% and 22% respectively). In general, the proportion of missing data increases over time; the differences between the consent groups in terms of missing data also increase. For example, for theft reported at age 18 years, the opt-in group at time of pilot linkage had 37% missing data, compared to 96% of the non-response group and 82% of the not sent for linkage group. The equivalent numbers by current consent status are 48%, 93% and 89% respectively.

Finally, we did not find a consistent pattern in self-reported anti-social and criminal behaviours when comparing participants who dissented to criminality linkage but did agree to at least one other linkage, compared to participants who did not dissent to any data linkage, or those who dissented to all data linkage options (but agreed to continue in ALSPAC). Comparison of these groups in terms of self-reported involvement with the criminal justice system was precluded by small numbers.

## Discussion

We completed a pilot record linkage in 2013 to determine the feasibility of linking an English population-based cohort study (ALSPAC) to official criminality records, and to inform whether a full linkage would be a worthwhile future endeavour in terms of creating a useful resource for research. The pilot was conditional on the extract being anonymous and not able to be linked to information on individual participants within the ALSPAC databank.

We first aimed to determine whether linkage to criminality records was acceptable to study participants, and whether there was sufficient criminality in the sample for research purposes. Criminal behaviour is a potentially sensitive area and so it was a positive finding that almost 900 participants with criminality record(s) enabled the linkage to happen through either explicit consent (in response to the opt-in request) or not objecting (in response to the opt-out fair processing campaign): out of a sample of 7,361 ALSPAC participants, 885 participants were linked to one or more criminality records held in the Police National Computer database. Further, our finding that - to date - only 4% of the sample have explicitly opted-out of linkage to criminality records supports the view that such linkage is acceptable to the majority of study participants. The group of participants who dissented to criminality linkage - but not to all linkage data sources - was small and within this group levels of self-reported criminality were low. With the available data we cannot determine if this sub-group of dissenters had engaged in a greater level of criminality compared to the rest of the sample and considered the research use of their criminality record to be sensitive. However, the proportion of participants who self-reported criminality and who did provide explicit consent could imply that participants trust the study to use these records appropriately for research. Whilst this could benefit from further research (ideally using mixed methods designs), this could inform future study designs and governance frameworks, and the considerations of ethical review boards.

In the sample of participants with criminal records, 4,000 convictions and cautions were recorded, many relating to serious crimes. If the linkage were repeated today, we would expect the number of criminal records to be substantially higher because (1) we now have permission to link to a larger sample and (2) there would now be more than 7 years of additional data. Therefore, we believe that there is a sufficient level of criminality in the ALSPAC sample for it to be a useful resource for crime-related research. However, it is unlikely that ALSPAC would have sufficient rates of less common crimes for these to be studied individually. We found criminal records from around the age of 12 years, but the majority of offences in our sample were committed later in adolescence. Therefore ALSPAC may not have sufficient numbers for research using linked criminality records at younger ages. Note that the PNC database is not ‘weeded’ (i.e. historical/spent convictions are not removed after a period of time) therefore this is not an explanation for the small amount of records at younger ages in our sample.

While all participants in the pilot were informed about the linkage and had not objected, only a sub-set of these had provided explicit consent. We found evidence suggesting different rates of self-reported criminal activity, and socio-demographic differences, according to consent status. Participants who explicitly consented to data linkage were more likely to be female, have higher socioeconomic status, lower levels of missing data and were less likely to self-report criminal behaviour. This pattern is similar to that found for general ALSPAC participation ^11^. This suggests that studies using only an opt-in sample may underestimate rates of criminal behaviour in the full study population. As such, it is necessary to consider the potential for selection bias when using a sample that relies on explicit opt-in consent status when designing linkage methodologies and considering the appropriateness of data sharing requests.

Finally, in order to inform which sources of crime data could be worthwhile pursuing for future linkage and research, we determined where the crimes committed by our sample took place. Our finding that the majority of offences in the pilot linkage were committed in the Avon and Somerset Police (A&SP) area, which has a similar geographical footprint to the ALSPAC recruitment area, suggests linkage to local police data held by A&SP, which contain more detail than that held in the national PNC, would capture most offences (at least to age 18). Working at a local level provides the opportunity to identify areas of research of local importance. However, at older ages criminal activity may become less geographically clustered, meaning centralised national records may be of increasing value.

### Strengths and limitations

There are several strengths and limitations to be considered in our pilot study. A strength was the wealth of data available on demographic measures and self-reported crime collected at multiple time-points, which allowed us to examine patterns in these variables by consent status. The ability to disaggregate our ‘sent for linkage’ group into those who actively opted-in and those who did not respond was a further strength as it enabled us to highlight the many differences between these groups. This is an important finding for other studies who are considering how to structure their consent campaigns, and will help inform the decision making of those reviewing this use of linked data in longitudinal studies. However, our evaluation is complicated by the fact that the sub-sample of participants included in the consent campaign, and those who were included in batch 1 versus batch 2, were not selected at random. This weakness is mitigated by the fact that this pilot study is intended to demonstrate viability rather than provide accurate association or prevalence estimates. Also, given that the sub-sample disproportionately included participants with strong levels of engagement, it can be hypothesised that this has led to an underestimate of recorded criminality within the sample.

The quality of data linkage relies on the accuracy of identifier records in both datasets (e.g. name, post code etc.). While ALSPAC’s administrative database is generally of good quality, it is likely to be out of date for some participants who are lost to follow-up. For the PNC data, the identifier database is known to have accuracy problems and includes pseudonyms, out of date information and duplicates ^14^. For example, individuals may report a false identity to the police. The linkage methodology used relied on deterministic matching that incorporates fuzzy parameters (i.e. where the requirement for all elements of the personal identifiers are relaxed in varying combinations). ALSPAC was not provided with a match quality score (which generates an estimate of the likelihood that two records relate to the same individual), which is counter to expectations that linkage quality estimates are transparent and available to the analyst ^17^.

It is also important to consider the quality of the data that is being linked from both sources and their potential limitations for answering questions in this research area. For PNC data, this depends on reliable and accurate testimony and record keeping. For ALSPAC records, the use of self-reported measures of criminal behaviours are vulnerable to social desirability bias, although the figures provided here illustrate that many participants are willing to report criminal and anti-social activity. Furthermore, drop out by participants may lead to bias.

Finally, as this pilot only produced a fully anonymous file, which cannot be linked to the wider ALSPAC dataset, there were limits to what could be included in this evaluation. For example, we could not examine relationships between official criminal records and the self-reported measures.

## Conclusions

We found differences in socio-demographic characteristics and rates of criminality according to the consent status of participants (i.e. explicit consent versus non-response to opt-out approaches), which suggest that methods of securing data must be considered carefully in future studies to reduce the risk of bias.

This pilot study illustrates that a full linkage of ALSPAC to crime records at an individual level would be a worthwhile future endeavour that would create a valuable resource for crime related research. Both local (Avon and Somerset) and national police records would be suitable for linkage, and linkage to both would be worthwhile. Advances in privacy preserving record linkage and ‘Trusted Research Environment’ secure research infrastructure and legislative changes (Digital Economy Act, DPA) may now enable linkage and the joint analysis of linked study-criminal record data under sufficiently controlled conditions to mitigate potential risks to confidentiality and help ensure that this form of data use is publicly acceptable. Individual-level linkage would enable direct comparisons between police-collected and self-reported criminal data, inform statistical strategies to account for missing data, and allow investigation of research questions related to the causal pathways to criminal behaviours using the wealth of life-course information collected by ALSPAC or other longitudinal studies. Once linked, these studies could provide valuable evidence to inform public health approaches to tackling crime.

## Data availability

### Underlying data

ALSPAC data access, including linked PNC data, is through a system of managed open access. The steps below highlight how to apply for access to ALSPAC data.

- Please read the ALSPAC access policy which describes the process of accessing the data in detail, and outlines the costs associated with doing so.
- You may also find it useful to browse the fully searchable research proposals database, which lists all research projects that have been approved since April 2011 including those using linked data.

For enquiries regarding linked data, please contact data-linkage@alspac.ac.uk.
