# Supplementary material for: The potential for linking cohort participants to official criminal records: a pilot study using the Avon Longitudinal Study of Parents and Children (ALSPAC)
Source: Wellcome Open Res. 2022 Sep 5;5:271. Originally published 2020 Nov 17. [Version 2] doi: 10.12688/wellcomeopenres.16328.2 (PMC9561538; doi:10.12688/wellcomeopenres.16328.2)
Supplement: Peer review history

# Peer review: Reviewer response for version 2

**DOI:** 10.21956/wellcomeopenres.19512.r52245

**Published:** 2022-10-13

**Authors:** Ben Matthews

**Affiliations:** 1 University of Stirling, Stirling, UK

This paper is much improved by the revisions - happy to approve.

Is the work clearly and accurately presented and does it cite the current literature?

Partly

If applicable, is the statistical analysis and its interpretation appropriate?

Yes

Are all the source data underlying the results available to ensure full reproducibility?

No

Is the study design appropriate and is the work technically sound?

Yes

Are the conclusions drawn adequately supported by the results?

Yes

Are sufficient details of methods and analysis provided to allow replication by others?

Yes

Reviewer Expertise:

Criminology.

I confirm that I have read this submission and believe that I have an appropriate level of expertise to confirm that it is of an acceptable scientific standard.

# Peer review: Reviewer response for version 2

**DOI:** 10.21956/wellcomeopenres.19512.r52246

**Published:** 2022-10-11

**Authors:** Kimberlie Dean

**Affiliations:** 1 School of Psychiatry, University of New South Wales, Sydney, Australia

Happy with revisions – no further comments.

Is the work clearly and accurately presented and does it cite the current literature?

Partly

If applicable, is the statistical analysis and its interpretation appropriate?

Partly

Are all the source data underlying the results available to ensure full reproducibility?

Partly

Is the study design appropriate and is the work technically sound?

Partly

Are the conclusions drawn adequately supported by the results?

Yes

Are sufficient details of methods and analysis provided to allow replication by others?

Yes

Reviewer Expertise:

NA

I confirm that I have read this submission and believe that I have an appropriate level of expertise to confirm that it is of an acceptable scientific standard.

# Peer review: Reviewer response for version 1

**DOI:** 10.21956/wellcomeopenres.17950.r42564

**Published:** 2021-02-24

**Authors:** Kimberlie Dean

**Affiliations:** 1 School of Psychiatry, University of New South Wales, Sydney, Australia

The submitted manuscript describes a pilot study in which a longitudinal cohort sample is linked to official criminal records. The submission is interesting and presents a useful contribution to the important and growing field of research using data arising from the linkage of cohorts/samples to large administrative datasets. The authors could consider the following points in making any amendments to the paper:

- There have been other studies employing a similar approach (i.e. linkage of criminal records/police data to longitudinal cohorts) that could have been cited (e.g. the NSW Child Development Study in Australia - Whitten *et al.* (2020) ^1^).
- The authors rightly identify the inability to actually link the criminal records obtained for the sample to the ALSPAC dataset - this is a very serious limitation of the approach (and really undermines the whole point of linking cohorts to administrative data - it would actually be better to obtain an anonymised randomly selected sample from the criminal records database and not link it to the ALSPAC cohort at all!); the authors should consider being clearer about this and explain why direct linkage is not possible; it is certainly managed in other settings (in some Australian states, a third party record-linkage organisation (e.g. the NSW Centre for Health Electronic Record Linkage or CHeReL) which undertakes the linkage to manage privacy/confidentiality).
- The most interesting aspect of the study is really the reported differences between those consenting to linkage, those not consenting and those not objecting (including in terms of self-reported criminal offending); the authors should consider formally testing these differences rather than just presenting descriptive statistics.

Is the work clearly and accurately presented and does it cite the current literature?

Partly

If applicable, is the statistical analysis and its interpretation appropriate?

Partly

Are all the source data underlying the results available to ensure full reproducibility?

Partly

Is the study design appropriate and is the work technically sound?

Partly

Are the conclusions drawn adequately supported by the results?

Yes

Are sufficient details of methods and analysis provided to allow replication by others?

Yes

Reviewer Expertise:

Forensic Mental Health, Psychiatric Epidemiology, Record linkage research.

I confirm that I have read this submission and believe that I have an appropriate level of expertise to confirm that it is of an acceptable scientific standard, however I have significant reservations, as outlined above.

## References

[1] : Children’s contact with police as a victim, person of interest and witness in New South Wales, Australia. *Australian & New Zealand Journal of Criminology* .2020;53(3) : 10.1177/0004865819890894 387-410 10.1177/0004865819890894

# Peer review: Reviewer response for version 1

**DOI:** 10.21956/wellcomeopenres.17950.r42177

**Published:** 2021-01-27

**Authors:** Ben Matthews

**Affiliations:** 1 University of Stirling, Stirling, UK

This is a very helpful paper describing a pilot project linking Avon Longitudinal Study of Parents and Children (ALSPAC) to Ministry of Justice (MoJ) records on criminal cautions and convictions.

 The paper is well-written, and provides useful information about the pilot linkage. I agree with the authors' conclusions that there are 'enough' people in ALSPAC with official cautions/convictions to make further linkage valuable analytically, and think that the further linkage discussed would be incredibly valuable for the reasons listed below. For such a future linkage MoJ including information on linkage error would be useful as the authors state.

 There are a few areas where I think the paper could be strengthened with some additional information about the datasets involved, for those not familiar with ALSPAC/PNC, which are listed below.

**Are all the source data underlying the results available to ensure full reproducibility?**

 I answered no, but this is not applicable given that it is a linkage study.

**Is the work clearly and accurately presented and does it cite the current literature?**

 There are two reasons I responded 'partly' to this question.

**1. Summary of criminological literature**

 From a criminologist's perspective, this linkage may be even more important than is emphasised in the paper. This is why I responded 'partly' to 'Is the work clearly and accurately presented and does it cite the current literature?'. 

 First, a cohort study with both self-reported and official offending data is particularly useful. Throughout the paper having both data sources is framed as a way to reduce measurement error in offending. This is a valid conceptual understanding of these two measures, but there is also a tradition in criminology, exemplified by the Edinburgh Study of Youth Transitions and Crime (McAra and McVie, 2010) ^1^, of understanding self-reported and official records measuring different constructs and analysing discrepancies in these data sources. There is a lot to be learned about the impacts of contact with the justice system itself from comparing the subsequent official criminal histories of people with similar levels of self-reporting but who do and do not come to the attention of the police. The capacity to make this kind of comparison in a contemporary cohort would be a real strength of linked ALSPAC/MoJ data.

 Second, one of the most important issues in contemporary criminology is understanding the international 'crime drop' over the last 25-30 years, and within this area of study one of the most informative but scarce resources are cohort studies. Comparing both self-reported and official offending records across different birth cohorts is a crucial part of understanding how this overall fall in crime is reflected in individual 'criminal careers' (e.g. Matthews, 2016; Payne and Piquero, 2020) ^2^^,^^3^, but the number of cohort studies which can be used for such comparisons is limited. As such, linking ALSPAC to MoJ records can provide an incredibly valuable resource to understand this important phenomenon.

 However, given that the primary audience for this paper might not be criminologists I don't necessarily think these points need to be discussed at length in the paper, but the first point in particular is important context. I leave it to the discretion of the authors as to whether to include the second point. 

 N.B. Both these potential benefits would come from a 'full' linkage of ALSPAC to MoJ records, rather than the pilot project described in this paper. 

**2. Description of the dataset/linkage**

 For someone who is not familiar with the ALSPAC dataset, it was tricky to reconcile the numbers in tables 3 and 4 with the study N listed on page 4. It would be helpful to add the total relevant N to these tables to make them a bit easier to understand. Similarly, for those not familiar with ALSPAC it would be helpful to provide a flow chart (or similar) which showed: 1. total ALSPAC N; 2. permission gained to link N; 3. successfully linked N etc.

 From Table 3 in particular, it would be helpful to clarify whether all ALSPAC children are asked the self-report questions at every age/are included in every ALSPAC questionnaire (for example, by including the N for each age). The terminology in Table 3 (CCQ, TF3... etc) does not map directly onto the questionnaire names included in the link at the bottom of the table, which makes finding more information about the questionnaires slightly cumbersome.

 It would also be useful to know how far back the PNC linkage goes, and whether Home Office Police National Computer (HOPNC) live database is ever 'weeded' (i.e. if historical/spent convictions are removed after a given period of time). Would we expect future linkage to list all convictions for the ALSPAC cohort (the age of criminal responsibility in England and Wales is 10 years old)? If PNC is not weeded it would be useful to say this explicitly in the text.

Is the work clearly and accurately presented and does it cite the current literature?

Partly

If applicable, is the statistical analysis and its interpretation appropriate?

Yes

Are all the source data underlying the results available to ensure full reproducibility?

No

Is the study design appropriate and is the work technically sound?

Yes

Are the conclusions drawn adequately supported by the results?

Yes

Are sufficient details of methods and analysis provided to allow replication by others?

Yes

Reviewer Expertise:

Criminology.

I confirm that I have read this submission and believe that I have an appropriate level of expertise to confirm that it is of an acceptable scientific standard, however I have significant reservations, as outlined above.

## References

[1] : Youth crime and justice: Key messages from the Edinburgh Study of Youth Transitions and Crime. *Criminology & Criminal Justice* .2010;10(2) : 10.1177/1748895809360971 179-209 10.1177/1748895809360971

[2] : Criminal careers and the crime drop in Scotland, 1989-2011: an exploration of conviction trends across age and sex.2016; Reference source

[3] : Developmental Criminology and the Crime Decline.2020; 10.1017/9781108882149 10.1017/9781108882149
